# Parawixin2 Protects Hippocampal Cells in Experimental Temporal Lobe Epilepsy

**DOI:** 10.3390/toxins10120486

**Published:** 2018-11-22

**Authors:** José Luiz Liberato, Lívea Dornela Godoy, Alexandra Olimpio Siqueira Cunha, Marcia Renata Mortari, Rene de Oliveira Beleboni, Andréia C. K. Fontana, Norberto Peporine Lopes, Wagner Ferreira dos Santos

**Affiliations:** 1 Neurobiology and Venoms Laboratory (LNP), Department of Biology, College of Philosophy, Sciences and Literature of Ribeirão Preto, University of São Paulo, Av. Bandeirantes, 3900, Ribeirão Preto, 14040-901 São Paulo, Brazil; jll@usp.br (J.L.L.); liveagodoy@usp.br (L.D.G.); alecunha@usp.br (A.O.S.C.); 2 Neuroscience Behavioral Institute (INEC), Av. do Café, 2450, Ribeirão Preto, 14050-220 São Paulo, Brazil; 3 Laboratory of Neuropharmacology, Department of Physiological Sciences, Institute of Biological Sciences, University of Brasília, DF 70910-900 Brasília, Brazil; mmortari@unb.br; 4 Department of Biotechnology/School of Medicine, University of Ribeirão Preto, Av. Costábile Romano, 2201, Ribeirão Preto, 14096-900 São Paulo, Brazil; rbeleboni@unaerp.br; 5 Department of Pharmacology and Physiology, Drexel University College of Medicine, 245 N. 15th Street, Philadelphia, PA 19102, USA; acm83@drexel.edu; 6 NPPNS, Department of Physics and Chemistry, College of Pharmaceutical Sciences of Ribeirão Preto, University of São Paulo, Ribeirão Preto, SP, Brazil, Av. do Cafe s/n, Ribeirão Preto, 14040-903 São Paulo, Brazil; npelopes@fcfrp.usp.br

**Keywords:** spider toxin, *Parawixia bistriata*, Parawixin2, GABA uptake inhibitor, temporal lobe epilepsy, pilocarpine model, hippocampal lost cells, neuroprotection

## Abstract

Epilepsy is considered as one of the major disabling neuropathologies. Almost one third of adult patients with temporal lobe epilepsy (TLE) do not respond to current antiepileptic drugs (AEDs). Additionally, most AEDs do not have neuroprotective effects against the inherent neurodegenerative process underlying the hippocampal sclerosis on TLE. Dysfunctions in the GABAergic neurotransmission may contribute not only to the onset of epileptic activity but also constitute an important system for therapeutic approaches. Therefore, molecules that enhance GABA inhibitory effects could open novel avenues for the understanding of epileptic plasticity and for drug development. Parawixin2, a compound isolated from *Parawixia bistriata* spider venom, inhibits both GABA and glycine uptake and has an anticonvulsant effect against a wide range of chemoconvulsants. The neuroprotective potential of Parawixin2 was analyzed in a model of TLE induced by a long-lasting Status Epilepticus (SE), and its efficiency was compared to well-known neuroprotective drugs, such as riluzole and nipecotic acid. Neuroprotection was assessed through histological markers for cell density (Nissl), astrocytic reactivity (GFAP) and cell death labeling (TUNEL), which were performed 24 h and 72 h after SE. Parawixin2 treatment resulted in neuroprotective effects in a dose dependent manner at 24 h and 72 h after SE, as well as reduced reactive astrocytes and apoptotic cell death. Based on these findings, Parawixin2 has a great potential to be used as a tool for neuroscience research and as a probe to the development of novel GABAergic neuroprotective agents.

## 1. Introduction

Temporal lobe epilepsy (TLE), the prevalent form of focal epilepsy, has the highest level of pharmacoresistance, since 30% of adult patients do not respond to current pharmacotherapy [1,2]. In TLE, the epileptiform activity is originated in the limbic system leading to a partial complex seizure [3], which in most cases are triggered by an initial neurological injury [4]. The injury process, most often due to a head trauma or a severe acute seizure known as Status Epilepticus (SE), is followed by an abnormal excitability in the limbic system, remarkably in the hippocampus [5,6]. Hippocampal sclerosis, characterized by neuronal loss, reactive astrocytosis, mossy fiber sprouting and glial proliferation [5,7,8], is a very common feature associated with TLE [9,10]. These findings have been based on data from biopsies of hippocampus from human TLE patients, as well as from histological observations in experimental models of TLE [10].

The pilocarpine-induced seizure in rats has been considered as a well-established model in TLE research due to great similarities with histopathological findings in TLE patients [7,11,12]. It has been shown that neuronal damage in the hippocampus occurs mostly during a specific time window, with severe neuronal loss at 24 h that reaches its peak at 72 h after the initial SE [13,14]. This TLE model has been widely used in the prospect of novel antiepileptic and neuroprotective compounds [15].

Previous studies have demonstrated that dysfunction in GABAergic inhibitory transmission is involved in many neuropathologies, including epilepsy [16]. It is well established that drugs acting on GABA uptake may provide effective means for protecting the brain against seizures [17]. However, only a few studies have investigated whether these drugs protect neurons against epileptogenic injury [18,19,20]. Therefore, the search for novel compounds that increase the GABA inhibitory activity may reveal new alternatives in the understanding and treatment of these pathologies [21,22,23].

GABA removal from the synaptic cleft can occur by diffusion or through sodium-dependent glial or neuronal transporters. Our group has isolated a compound from the venom of the spider *Parawixia bistriata* denominated as Parawixin2 (formerly FrPbAII), which has been shown to be a potent inhibitor of GABA and glycine uptakes [24].

Parawixin2 was shown to suppress tonic-clonic seizures induced by bicuculine [25] pilocarpine, kainic acid, pentylenetetrazol, and picrotoxin [26], as well as to decrease the incidence and severity of seizures induced by bicuculine in the Area Tempestas [27]. Moreover, Parawixin2 blocked seizures in an animal model of chronic epilepsy, the PTZ-induced kindling [28] and exerted a marked neuroprotective effect in all retina cell layers in a model of retinal ischemia [24].

Furthermore, in a recent study, Godoy [20] has found that Parawixin2 reduced spontaneous recurrent seizures (SRS) frequency and protected CA3 and DG hippocampal regions against neuronal death in a chronic temporal lobe epilepsy model. Noteworthy, Parawixin2 treatment resulted in a significant neuroprotective effect in preserving parvalbuminergic neurons, thus suggesting potential seizure control in antiepileptic treatment [20].

In light of these findings, the aim of this work was to analyze potential neuroprotective effects of Parawixin2 in time specific windows of neurodegenerative processes induced by the pilocarpine model of experimental TLE.

## 2. Results

### 2.1. Behavioral Characteristics of Status Epilepticus

Injection of pilocarpine (i.c.v.) induced SE in 96 out of 120 rats (80%), which exhibited limbic seizures characterized by orofacial automatisms, hyper salivation, forelimb clonus, and localized myoclonus, followed by rearing, rearing and falling, and loss of postural control (score 5 of the Racine scale).

### 2.2. Histopathologic Analysis of Neuronal Tissues

**Figure 1:**
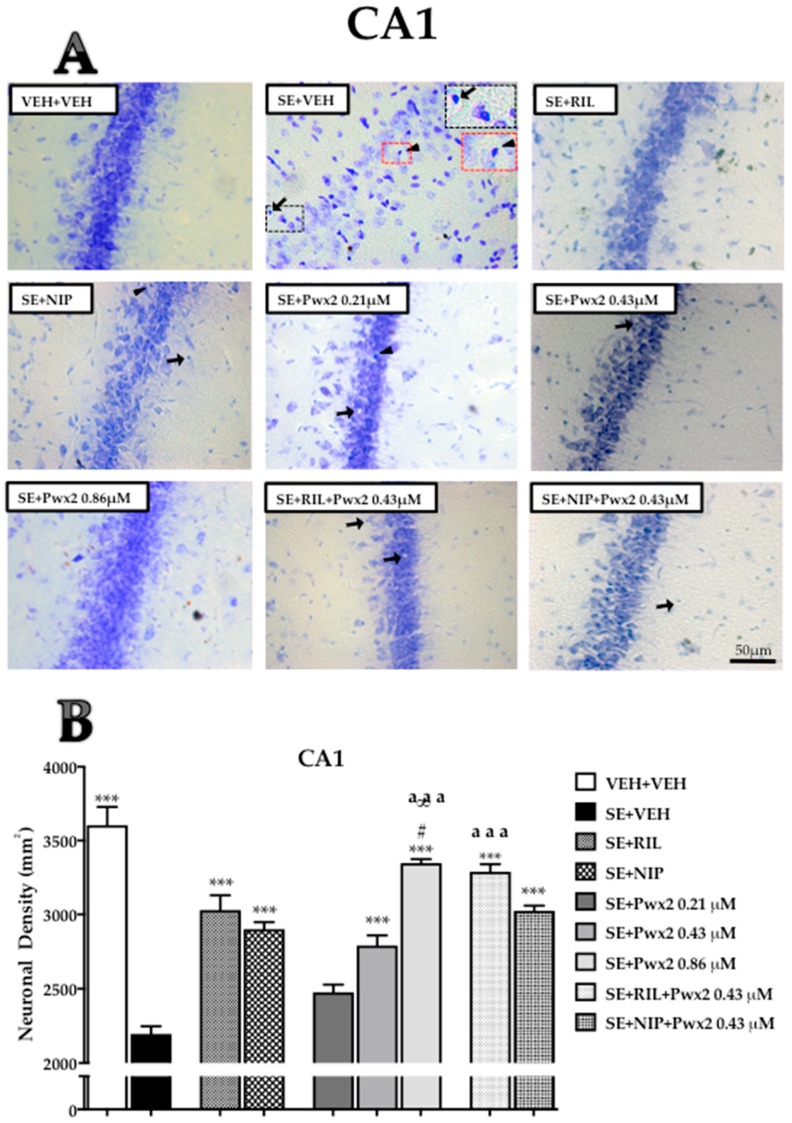
Hippocampal coronal sections showing Nissl-stained of the CA1 pyramidal cell layer assessed 24 h after pilocarpine-induced Status Epilepticus (SE). (**A**) Arrows point to pyknotic nuclei while arrowheads point to vacuolized cells. (**B**) Quantitative analysis of neuronal densities on the CA1 pyramidal cell layer. Data represents mean of neurons per mm^2^ ± standard error of the mean (SEM) of pyramidal cell layers of CA1. *** *p* < 0.001 compared to the SE + VEH group (rats submitted to SE and treated with vehicle); # *p* < 0.05, in comparison to the SE + RIL group (rats submitted to SE and treated with riluzole); ∞ *p* < 0.05, relate to SE + NIP (rats submitted to SE and treated with nipecotic acid); aaa *p* < 0.001 compared to SE + Pwx2 0.43 µM (rats submitted to SE and treated with Parawixin2 0.43 μM). Scale bar: 50 μM.

**Figure 2:**
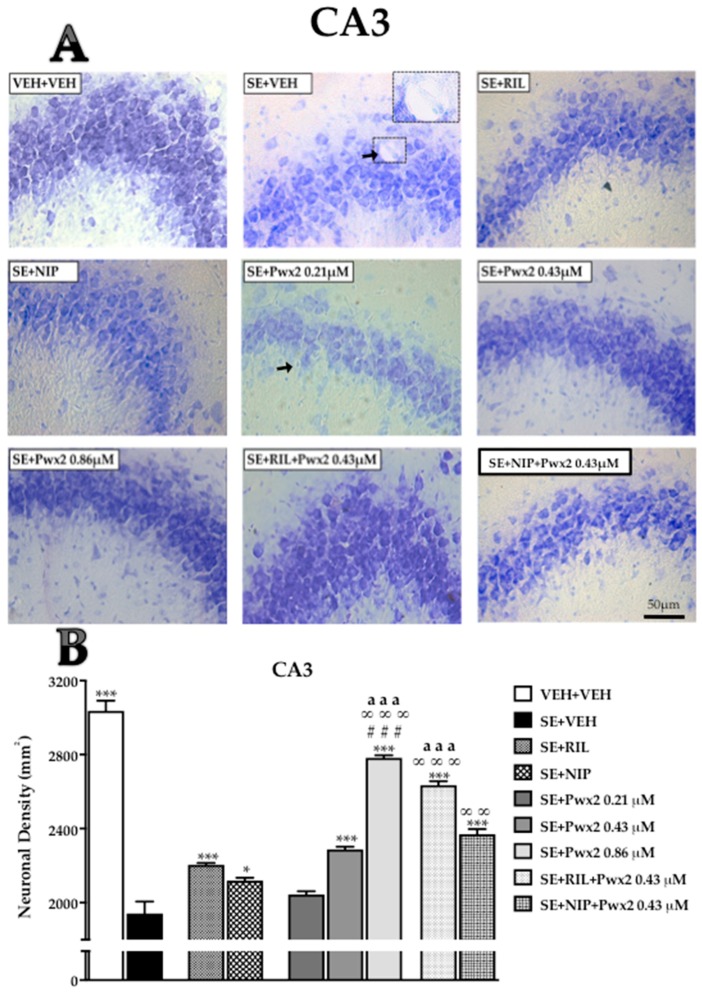
Hippocampal coronal sections showing Nissl-stained in the CA3 pyramidal cell layer assessed 24 h after pilocarpine-induced SE. (**A**) Arrows point to pyknotic nuclei while arrowheads point to vacuolized cells. (**B**) Quantitative analysis of neuronal densities on the CA3 pyramidal cell layer. Data represents mean of neurons per mm^2^ (± SEM) of pyramidal cell layers of CA3). * *p* < 0.05, and *** *p* < 0.001 compared to the SE + VEH group; * *p* < 0.05 ### *p* < 0.001 in comparison to the SE + RIL group; ∞ ∞ *p* < 0.01 and ∞ ∞ ∞ *p* < 0.001 relate to SE + NIP; aaa *p* < 0.001 compared to SE + Pwx2 0.43 µM. Scale bar: 50 μM.

**Figure 3:**
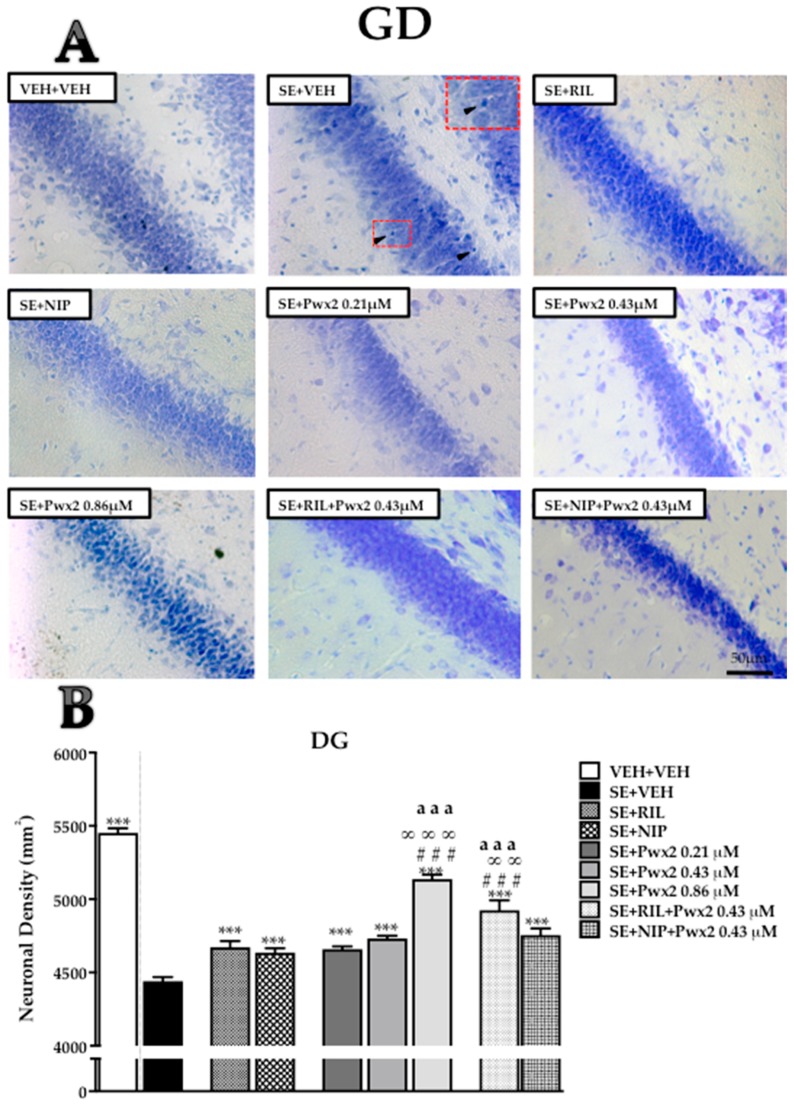
Hippocampal coronal sections showing Nissl-stained in the dentate gyrus granular cell layer assessed 24 h after pilocarpine-induced SE. (**A**) Arrows point to pyknotic nuclei while arrowheads point to vacuolized cells. (**B**) Quantitative analysis of neuronal densities on dentate gyrus (DG) granular cells. Data represents mean of neurons per mm^2^ (± SEM) of DG granular cells. *** *p* < 0.001 compared to the SE + VEH group; # # # *p* < 0.001 in comparison to the SE + RIL group; ∞ ∞ *p* < 0.01 and ∞ ∞ ∞ *p* < 0.001 relate to SE + NIP; aaa *p* < 0.001 compared to SE + Pwx2 0.43 µM. Scale bar: 50 μM.

Qualitative analysis of brain sections from the SE + VEH (rats submitted to SE and treated with vehicle) group revealed severe damage throughout the dorsal hippocampus 24 h after SE. All hippocampal areas examined of SE + VEH rats exhibited shrunken neurons, nuclear pyknosis, cytoplasmic vacuolar degeneration, and extensive gliosis, mostly in CA1 (Figure 1) and CA3 (Figure 2), which also exhibited disorganization of pyramidal cell layers. The hippocampal tissue of rats treated with 0.86 μM Parawixin2 exhibited pyramidal organized cell layers and fewer neurons with altered morphology than all other experimental groups (Figure 1A, Figure 2A and Figure 3A). Histological abnormalities were also observed in the granular cell layer of the dentate gyrus (DG), however in a lesser extent than in CA1 and CA3.

The qualitative analyses were based on representative images of the quantitative results, which are described below by each hippocampal sublayer.

After 24 h of SE, analysis of mean cell density revealed a significant difference between groups in CA1 (Figure 1B) [F (8, 64) = 52.03, *p* < 0.001)]. The SE + VEH group presented an evident cellular loss compared to VEH + VEH (*p* < 0.001). Additionally, both SE + Pwx2 (rats submitted to SE and treated with Parawixin2) 0.21 and 0.43 μM groups, SE + RIL (rats submitted to SE and treated with riluzole) and SE + NIP (rats submitted to SE and treated with nipecotic acid) were significantly different from VEH + VEH (*p* < 0.001). However, when pharmacological treatments were compared to the SE + VEH group, all resulted in significantly reduced cell loss (*p* < 0.0001) except for SE + Pwx2 0.21 μM group.

In order to evaluate which drugs presented more neuroprotective potential, we compared all effective treatments with each other. This procedure was applied to all following sublayers. We observed that SE + Pwx2 0.86 μM resulted in significantly higher cell density when compared to SE + RIL (*p* < 0.05), SE + NIP (*p* < 0.05) and SE + Pwx2 0.43 μM (*p* < 0.001).

Groups that received co-treatments of Pwx2 with RIL and NIP also significantly reduced cell loss when compared to SE + VEH (*p* < 0.0001), but only SE + RIL + Pwx2 showed significantly higher mean cell density compared to SE + Pwx2 0.43 μM (*p* < 0.001).

In the CA3 subregion we observed a very similar pattern as CA1 [F (8, 64) = 84.41, *p* < 0.0001)]. Additionally, the SE + VEH group demonstrated an intense cell loss compared to VEH + VEH (*p* < 0.0001). Again, all treatments resulted in a reduction in the neurodegeneration process compared to the SE + VEH group (*p* < 0.001), except for the SE + Pwx2 0.21 μM group. Additionally, as in CA1, treatment with SE + Pwx2 0.86 μM resulted in a higher neuronal density, when compared to SE + RIL (*p* < 0.0001), SE + NIP (*p* < 0.001) and SE + Pwx2 0.43 μM (*p* < 0.001). Groups that received co-treatments with SE + RIL + Pwx2 and SE + NIP + Pwx2 also showed significantly reduced cell loss when compared to SE + VEH (*p* < 0.001). Both SE + RIL+ Pwx2 (*p* < 0.001) and SE + NIP + Pwx2 (*p* < 0.01) differed from SE + NIP. Moreover, SE + RIL + Pwx2 showed significantly higher cell density compared to SE + Pwx2 0.43 μM (*p* < 0.001).

As observed in the qualitative analysis, the DG granular cell layer seems to be more resistant than CA1 and CA3 to the insult, but we still observed a significant cell loss with SE [F (8, 64) = 85.40, *p* < 0.0001]. Once more, SE + VEH showed a significant reduction in comparison to VEH + VEH (*p* < 0.001). In this sublayer, all treatments significantly decreased cell loss induced by SE (*p* < 0.001). Yet again, it was observed that SE + Pwx2 0.86 μM had a higher cell density compared to SE + RIL (*p* < 0.001), SE + NIP (*p* < 0.001) and SE + Pwx2 0.43 μM (*p* < 0.001). Co-treatments of Pwx2 with RIL and NIP resulted in reduced cell loss when compared to SE + VEH (*p* < 0.001), as in CA1 only SE + RIL + Pwx2 showed significantly higher mean cell density compared to SE + Pwx2 0.43 μM (*p* < 0.001).

### 2.3. Immunohistochemical Analysis of TUNEL-Labeled (Terminal Deoxynucleotidyl Transferase Immunostaining for Biotin-dUTP-Nick-End-Labeling) Cells

**Figure 4:**
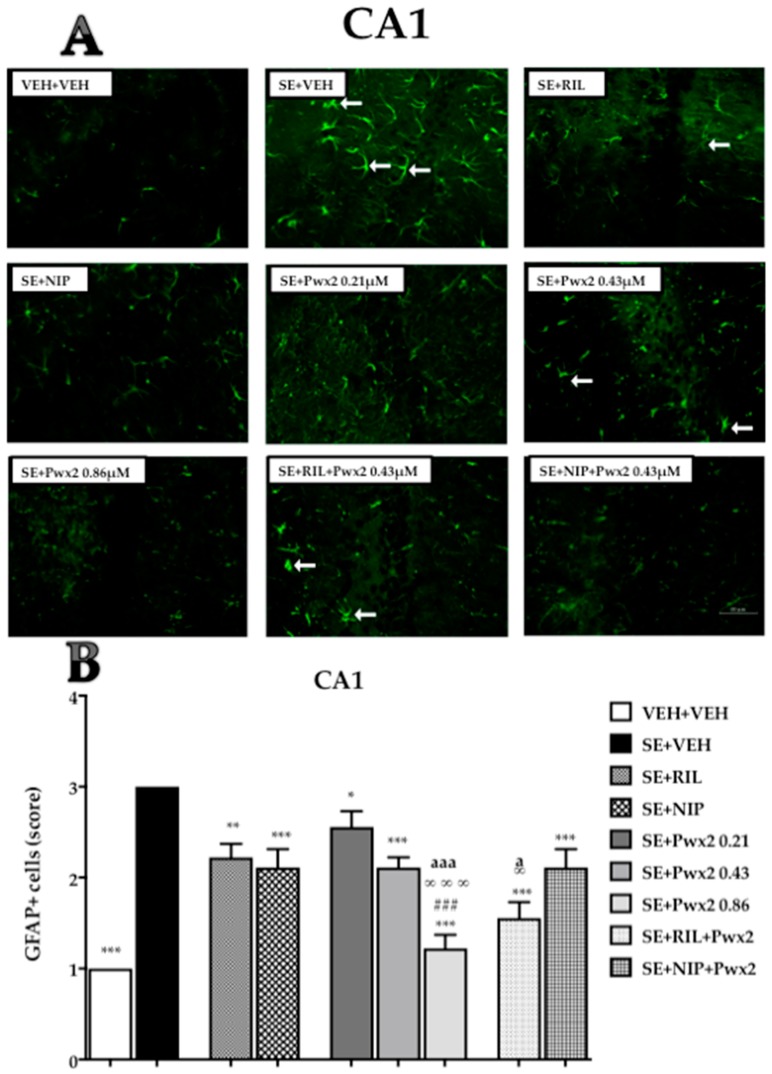
TUNEL positive cells showing programmed cellular death process in the CA1 pyramidal cell layer 24 h after long-lasting pilocarpine-induced SE. (**A**) Arrows point TUNEL+ neurons. Data represent the mean of semi-quantitative analysis of TUNEL+ neurons (**B**). * *p* < 0.05, ** *p* < 0.01 and *** *p* < 0.001 compared to the SE + VEH group; ### *p* < 0.001 in comparison to the SE + RIL group; ∞ *p* < 0.05 and ∞ ∞ ∞ *p* < 0.001 relate to SE + NIP; a *p* < 0.001 and aaa *p* < 0.001 compared to SE + Pwx2 0.43 µM. Scale bar: 50 μM.

Semi-quantitative analysis of TUNEL-labeled cells in the CA1 hippocampal region [F (8, 46) = 6.52; *p* < 0.001] showed rats of VEH + VEH had an insignificant level of staining (Table 1 and Figure 4). Conversely, the SE + VEH group exhibited a moderate degree of TUNEL+ cells compared to VEH + VEH (*p* < 0.001), and the treatment with Pwx2 0.86 μM was the only one that significantly reduced TUNEL-labeled cells (*p* < 0.05) (Figure 4).

**Figure 5:**
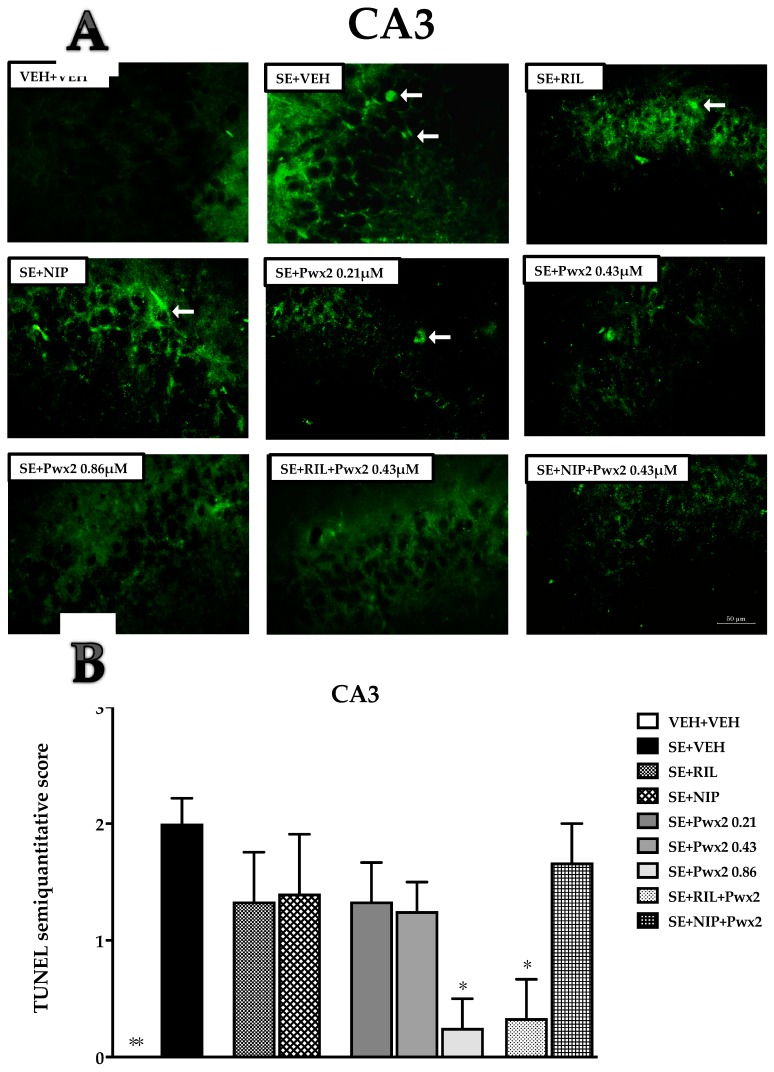
TUNEL positive cells showing programmed cellular death process in the CA3 pyramidal cell layer 24 h after long-lasting pilocarpine-induced SE. (**A**) Arrows point to TUNEL+ neurons. Data represent the mean of semi-quantitative analysis of TUNEL+ neurons (**B**). * *p* < 0.05, and ** *p* < 0.01 compared to the SE + VEH group. Scale bar: 50 μM.

Additionally, data from the CA3 pyramidal cell layer [F (8, 41) = 3.98, *p* = 0. 0026] shows that the SE + VEH group presents more TUNEL+ labeling in comparison to VEH + VEH (*p* < 0.01). In this sublayer, only Pwx2 0.86 μM and the combination of RIL and Pwx2 (SE + RIL+ Pwx2) treatments were effective in reducing TUNEL-labeling when compared to SE + VEH (*p* < 0.05) (Table 1 and Figure 5).

**Figure 6:**
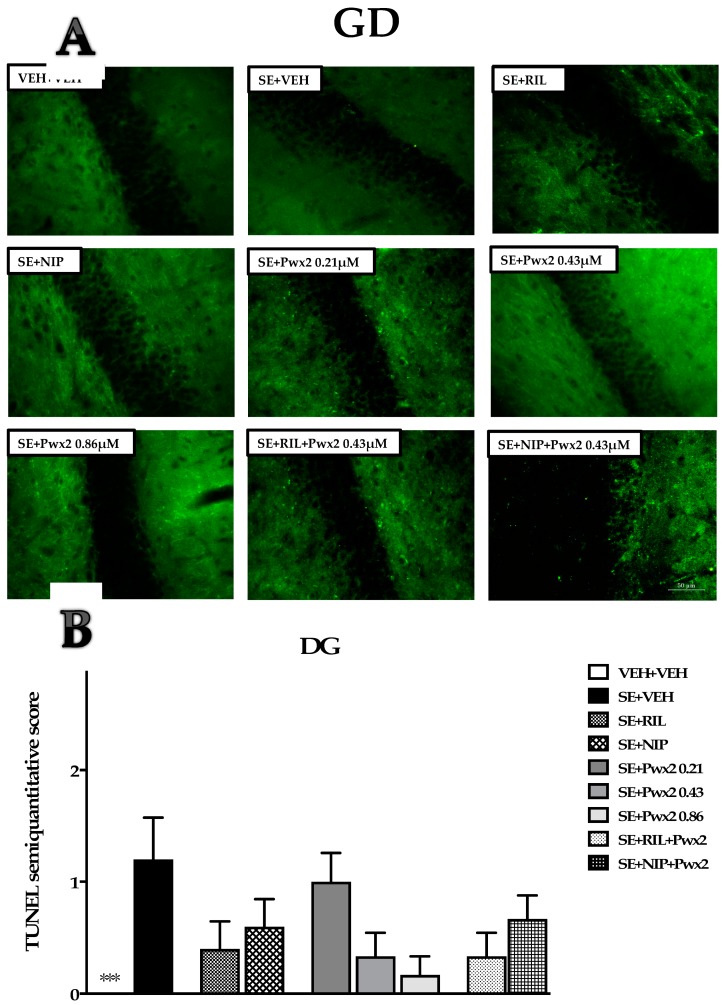
TUNEL positive cells in programmed cellular death process in the Dentate Gyrus (DG) granular cell layer 24 h after long-lasting pilocarpine-induced SE. (**A**) Arrows point to TUNEL+ neurons. Data represent the mean of semi-quantitative analysis of TUNEL+ neurons (**B**). *** *p* < 0.001 compared to the SE + VEH group. Scale bar: 50 μm.

Similar to the Nissl data, in the DG region, a lower level of TUNEL reactive cells was observed in all groups submitted to SE (Table 1 and Figure 6) and there was no difference between the treatments [F (4, 12) = 2.455; *p* = 0.1024].

### 2.4. GFAP Immunofluorescence Detection

**Figure 7:**
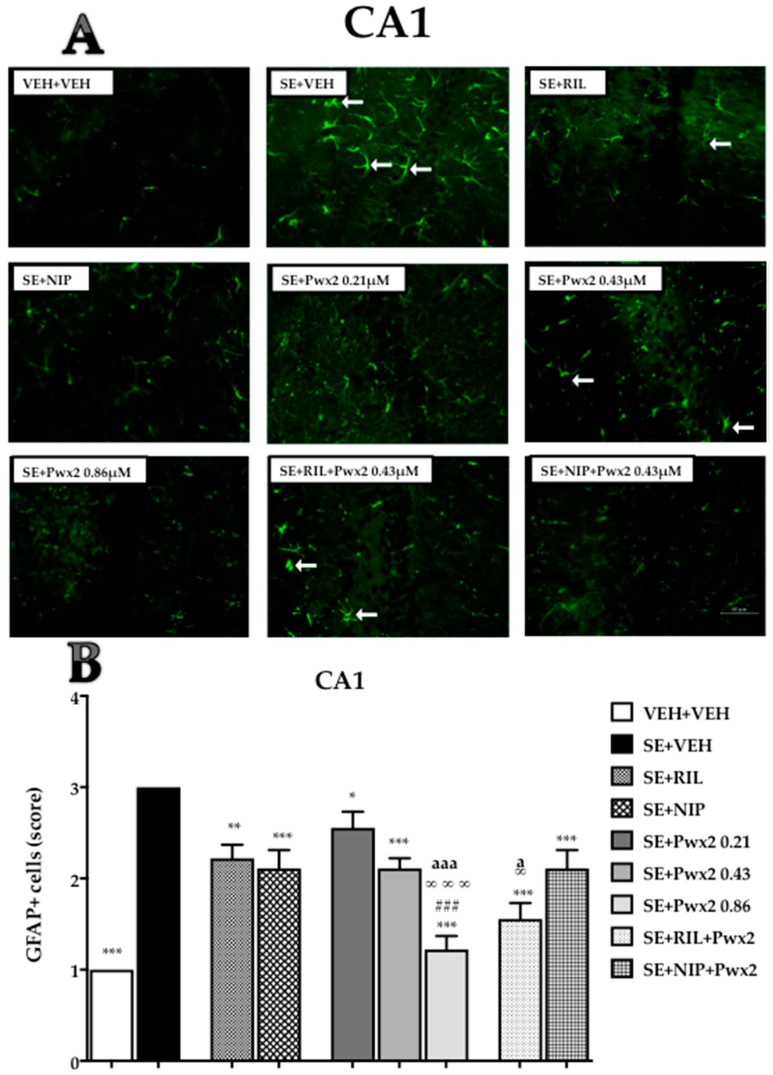
Quantification of glial fibrillary acidic protein (GFAP) staining cells showing labeled astrocytes in CA1 pyramidal cell layer 24 h after long-lasting pilocarpine-induced SE. (**A**) Arrows point to reactive astrocytes. (**B**) Data are shown as mean of cells GFAP+. * *p* < 0.05, ** *p* < 0.01 and *** *p* < 0.001 compared to the SE + VEH group; * *p* < 0.05, ** *p* < 0.01 and *** *p* < 0.001 compared to the SE + VEH group; # *p* < 0.05, and ### *p* < 0.001 in comparison to the SE + RIL group; ∞ *p* < 0.05, and ∞∞∞ *p* < 0.001 relate to SE + NIP; a *p* < 0.05, and aaa *p* < 0.001 compared to SE + Pwx2 0.43 µM.

GFAP-immunoreactivity (GFAP+) in CA1 was affected by treatments [F (10, 88) = 21.65; *p* < 0.001] which can be observed by a more pronounced number and reactivity in groups submitted to SE. Comparing VEH + VEH to the SE + VEH group, we observed an intense GFAP+ immunolabelling (score 3; *p* < 0.05) (Figure 7). All treatment groups (SE + RIL, SE + NIP, SE + Pwx2 0.43 μM, SE + Pwx2 0.86 μM, SE + NIP + Pwx2 and SE + RIL+ Pwx2) showed a reduction in GFAP+, when compared to the SE + VEH group (*p* < 0.001 for all), reaching moderate to low scores of GFAP+ cells (Figure 7).

Treatment with Pwx2 0.86 μM exhibited the best outcome, which was almost similar to VEH + VEH staining. Additionally, this treatment differed from SE + Pwx2 0.43 µM, SE + RIL and SE + NIP groups (*p* < 0.001). Furthermore, SE + RIL+ Pwx2 0.43 μM (*p* < 0.001) showed a significant reduction in GFAP+ cells compared to both SE + Pwx2 0.43 µM and SE + RIL groups.

**Figure 8:**
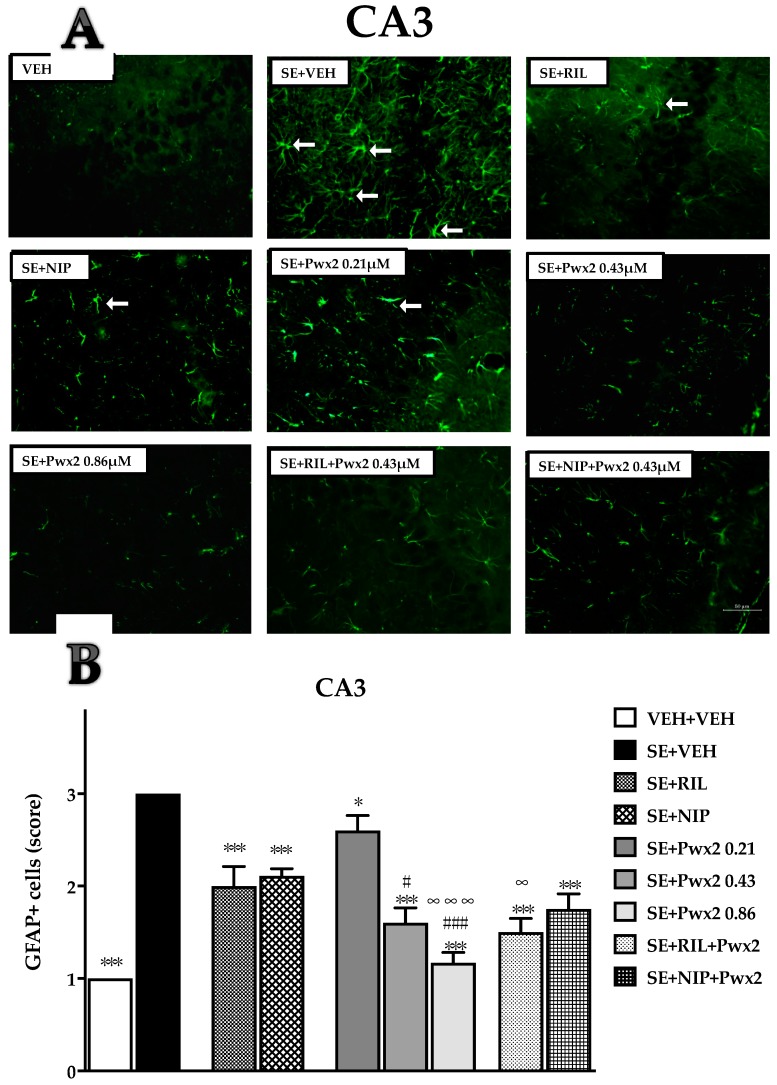
Quantification of glial fibrillary acidic protein (GFAP) staining cells showing labeled astrocytes in CA3 pyramidal cell layer after long-lasting pilocarpine-induced SE. (**A**) Arrows point to reactive astrocytes. (**B**) Data represent mean of cells GFAP+ 24 h after SE. * *p* < 0.05, ** *p* < 0.01 and *** *p* < 0.001 compared to the SE + VEH group; * *p* < 0.05, ** *p* < 0.01 and *** *p* < 0.001 compared to the SE + VEH group; # *p* < 0.05, and ### *p* < 0.001 in comparison to the SE + RIL group; ∞ *p* < 0.05, and ∞ ∞ ∞ *p* < 0.001 relate to SE + NIP.

Similar results were observed in the CA3 subregion [F (8, 99) = 30.13; *p* < 0.001], whereas compared to SE + VEH, a moderate score of GFAP+ cells was observed in SE + RIL (*p* < 0.001), SE + NIP (*p* < 0.001), SE + Pwx2 0.21 μM (*p* < 0.05), and SE + Pwx2 0.43 μM (*p* < 0.001). Co-treatments groups SE + RIL+ Pwx2 and SE + NIP+ Pwx2 also resulted in a reduced score of GFAP+ cells in comparison to SE + VEH (Figure 8). As observed in the CA1 region, the treatment with Pwx2 0.86 μM exhibited the lower level of GFAP+ immunolabelling in comparison to SE + VEH (*p* < 0.001), which also significantly differed from the SE + RIL and SE + NIP groups (Figure 8).

**Figure 9:**
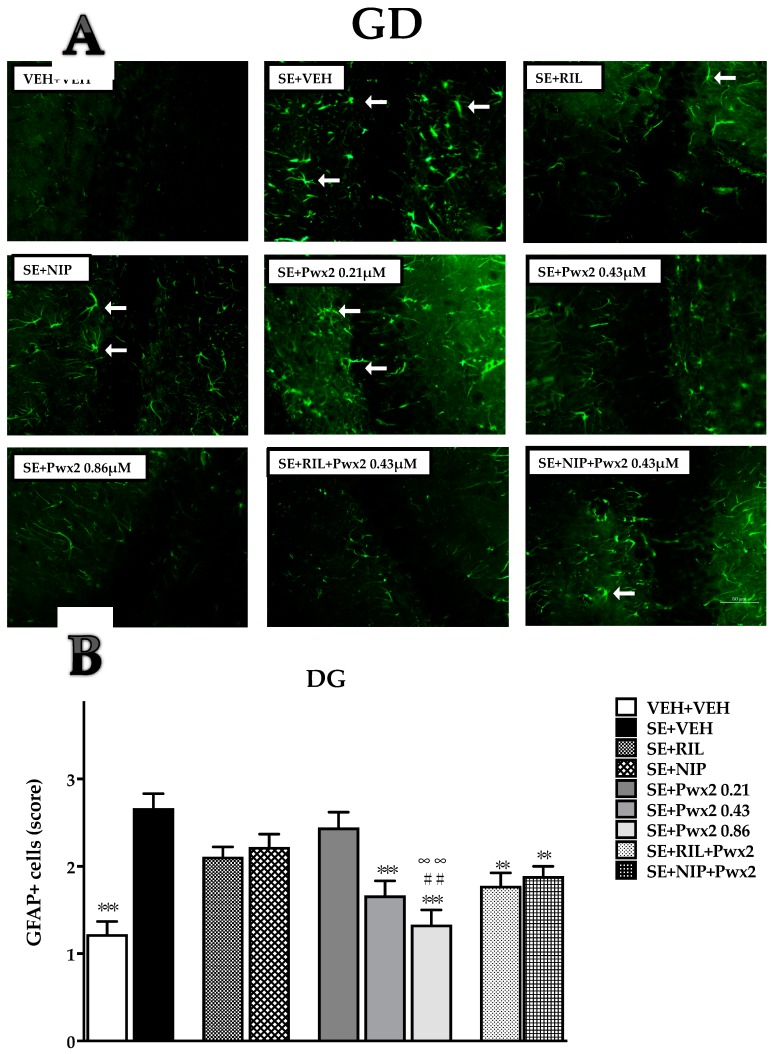
Quantification of glial fibrillary acidic protein (GFAP) staining cells showing labeled astrocytes in Dentate Gyrus assessed 24 h after long-lasting pilocarpine-induced SE. (**A**) Arrows point to reactive astrocytes. (**B**) Data represent mean of cells GFAP+. ** *p* < 0.01 and *** *p* < 0.001 compared to the SE + VEH group; * *p* < 0.05, ** *p* < 0.01 and *** *p* < 0.001 compared to the SE + VEH group; ## *p* < 0.01 in comparison to the SE + RIL group; ∞∞ *p* < 0.01 relate to SE + NIP.

In the DG region, data shows that only SE + Pwx2 0.43 μM (*p* < 0.001), SE + RIL + Pwx2 and SE + NIP + Pwx2 (*p* < 0.01) groups exhibit a significant reduction in the score of GFAP+ cells when compared to SE + VEH (F (8, 72) = 10.36; *p* < 0.001) (Figure 9). Once more, the treatment with Pwx2 0.86 μM was the most effective in decreasing GFAP+ reactivity induced by SE (*p* < 0.001) that also significantly differed from SE + RIL and SE + NIP (*p* < 0.01) (Figure 9).

### 2.5. Comparison of Histopathological Biomarkers at 24 h and 72 h after SE

**Figure 10:**
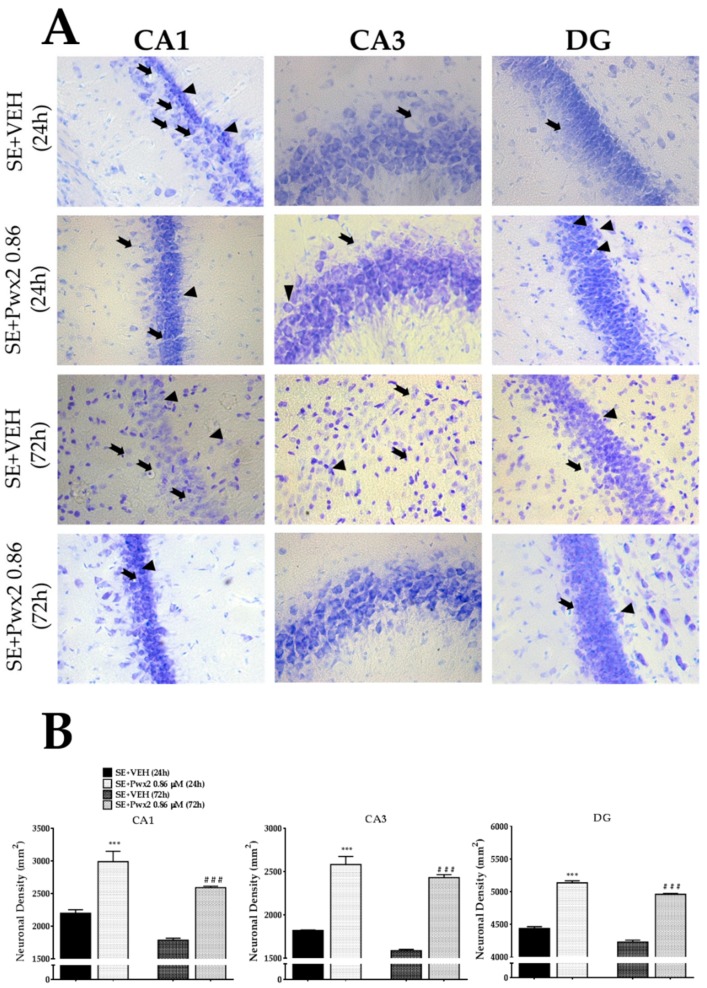
Brain sections of rats in coronal plane showing neurons and glia Nissl-stained of the CA1, CA3 and DG assessed 24 h and 72 h after pilocarpine-induced SE. (**A**). Arrows point to pyknotic nuclei while arrowheads point to vacuolized cells. (**B**) Quantitative analysis of neuronal densities on the hippocampal regions of Wistar rats 24 h and 72 h after SE termination of pilocarpine-induced SE. Data represents mean of neurons per mm^2^ (± SEM) of pyramidal cell layers of CA1, CA3 and the granular cell layer of dentate gyrus. * *p* < 0.05; and *** *p* < 0.001, in comparison to the SE + VEH 24 h group. ### *p* < 0.001, in comparison to the SE + VEH 72 h group (one-way ANOVA and Student–Newman–Keuls as post-test). Scale bar: 50 μM.

As previously observed, pilocarpine-induced cell death still can be observed 72 h after SE (Figure 10). Thus, two-way ANOVA analysis revealed a significant effect of time in the neurodegenerative process in CA1 [F (1, 31) = 37.18; *p* < 0.0001] which was confirmed by a significant increase in cell loss in CA1 of the SE + VEH 72 h group (*p* < 0.05), when compared to the SE + VEH 24 h group [F (5, 39) = 27.64; *p* < 0.001] (Figure 10). Additionally, data showed a significant effect for treatment [F (1, 31) = 9.59; *p* = 0.0041] and interaction [F (1, 26) = 20.71, *p* = 0.0001]. We found that the highest dose of Parawixin2 (0.86 μM), which had previously shown the best neuroprotective effects in 24 h analysis, also proved to be neuroprotective even 72 h after onset of SE in CA1 (*p* < 0.001), in comparison to SE + VEH 72 h (Figure 10).

We observed equivalent results in CA3 for time [F (1, 24) = 252.4; *p* < 0.001], treatment [F (1, 24) = 243.6; *p* < 0.001] and interaction [F (1, 24) = 10.98; *p*= 0.0029]. Additionally, in CA3, the SE + VEH 72 h group shows a significative reduction of cells density (*p* < 0.001) compared to SE + VEH 24 h. Furthermore, the treatment with Pwx2 0.86 μM protects this cell layer against cell loss (*p* < 0.001) (Figure 10).

As observed in the other hippocampal regions analyzed, the reduction in neuronal density throughout time was also observed in DG [F (1, 24) = 49.93; *p* < 0.001]. As in CA1 and CA3, SE + VEH 72 h after SE was significantly reduced in comparison to the SE + VEH 24 h group (*p* < 0.001). Again, the treatment [F (1, 24) = 689.2; *p* < 0.001] factor was significantly different, but not for the interaction factor [F (1, 24) = 0.3780; *p* = 0.5444]. The cell density in DG of the SE + Pwx2 0.86 μM 72 h group was significantly higher than SE + VEH 72 h (*p* < 0.001) (Figure 10).

**Figure 11:**
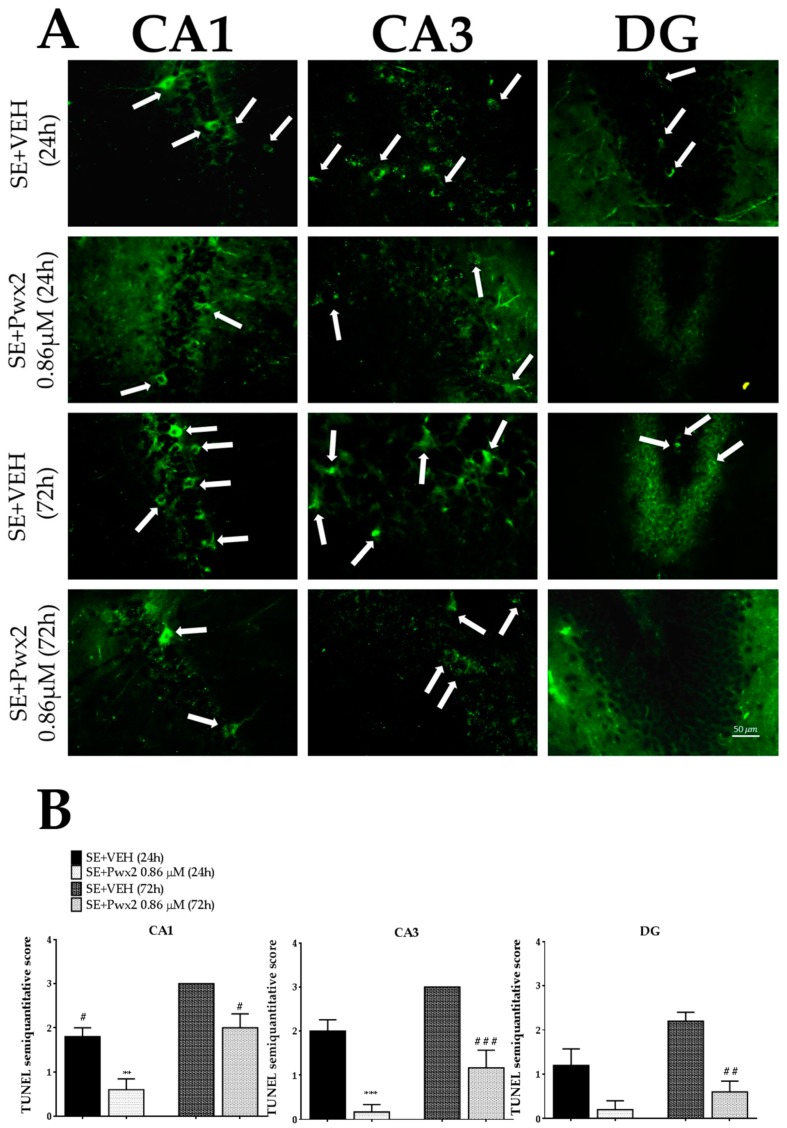
TUNEL positive cells showing programmed cellular death process in CA1, CA3 and DG 24 h and 72 h after long-lasting pilocarpine-induced SE. (**A**) Arrows point TUNEL+ neurons. Data represent the mean of semi-quantitative analysis of TUNEL+ neurons (**B**). ** *p* < 0.01 and *** *p* < 0.001 compared to the SE + VEH (24 h) group; # *p* < 0.05, ## *p* < 0.01 and ### *p* < 0.001 in comparison to the SE + VEH (72 h) group. Scale bar: 50 μM.

Moreover, Nissl data was corroborated by TUNEL as we observed a time dependent factor in CA1 [F (8, 46) = 6.519; *p* < 0.001], CA3 [F (8, 30) = 3.986; *p* < 0.001] and DG [F (8, 41) = 2.746; *p* = 0.0159]. It demonstrated a marked increase in the SE + VEH 72 h number of TUNEL+ cells 72 h after SE in CA1 (*p* < 0.001), and CA3 (*p* < 0.001), in comparison to the SE + VEH 24 h group (Figure 11). Additionally, treatment was effective in reducing the number of TUNEL positive cells in CA1 [F (3, 18) = 43.41; *p* < 0.001], CA3 [F (3, 18) = 24.24; *p* < 0.001] and DG [F (3, 18) = 26.02; *p* < 0.0001] in comparison to the SE + VEH 72 h group (*p* < 0.05, *p* < 0.001 and *p* < 0.01 in CA1, CA3 and GD, respectively).

**Figure 12:**
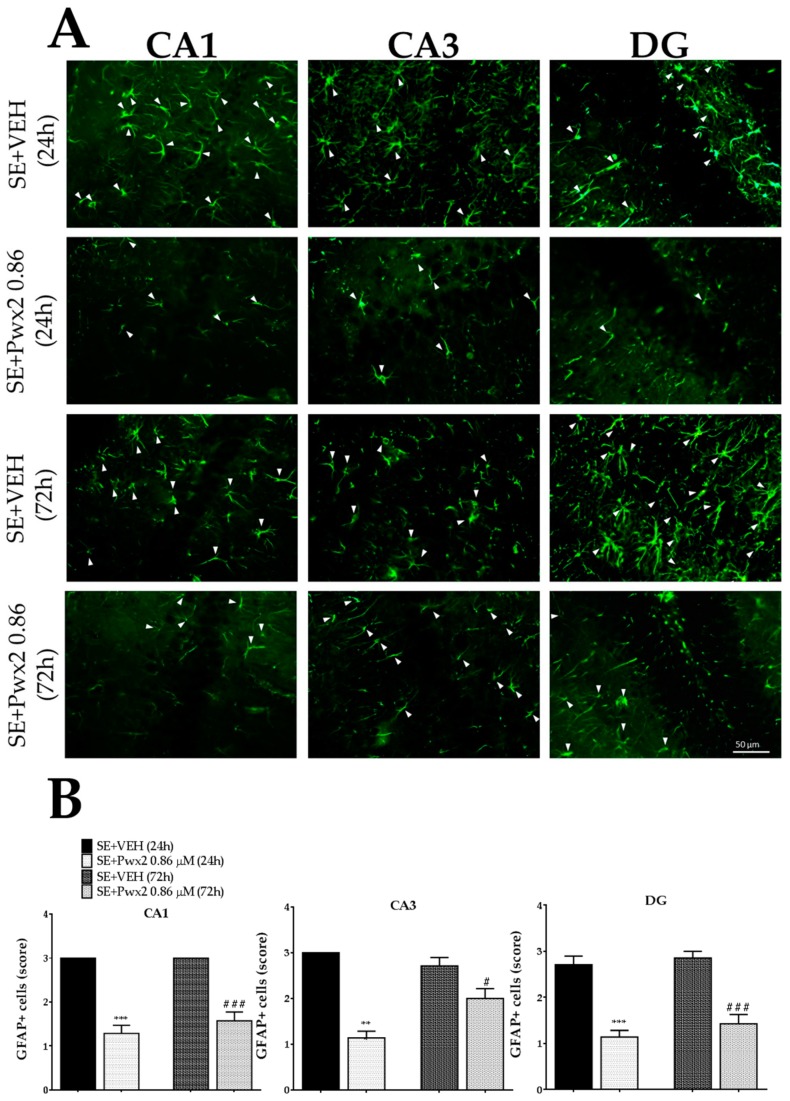
Reactive astrocytes in hippocampal formation 24 h and 72 h after long-lasting pilocarpine-induced SE. Arrows point to GFAP reactive cells (**A**). Data represent the mean of semi-quantitative analysis of GFAP+ (**B**). ** *p* < 0.01 and *** *p* < 0.001 compared to the SE + VEH (24 h) group; # *p* < 0.05, ## *p* < 0.01 and ### *p* < 0.001 in comparison to the SE + VEH (72 h) group. Scale bar: 50 μM.

Quantification of GFAP reactive cells showed no difference between the groups SE + VEH (24 h) and SE + VEH (72 h) in all hippocampal subfields analyzed. However, Parawixin2 administration 72 h after SE resulted in a reduction in the level of GFAP+ cells in CA1 [F (3, 18) = 43.41; *p* < 0.001], CA3 [F (3, 18) = 24.24; *p* < 0.001] and DG [F (3, 18) = 26.02; *p* < 0.0001] in comparison to the SE + VEH 72 h group (Figure 12).

## 3. Discussion

In the present study, we investigated the neuroprotective effects of Parawixin2, a GABA and glycine uptake inhibitor [24] in the intracerebral pilocarpine TLE model. The pilocarpine-induced SE animal model has been proven to be a very important tool for studying human TLE and for the evaluation of new antiepileptic and neuroprotective drugs, as it has some neuropathological features very similar to those observed in the hippocampal tissue resected from TLE patients [29,30,31]. As reported in previous studies, profound levels of cell death are observed in the pyramidal cell layers of CA1 and CA3, as well as in the granule cell layer of DG and in the hilus of the hippocampus after pilocarpine-induced SE in rats [12,32,33]. In this model, TUNEL immunolabeling has been extensively used as an indicator of programmed cell death [34].

Our data show a remarkable neuroprotective effect of Parawixin2, in a dose-dependent manner with a more pronounced effect in the group treated with 0.86 μM, against prolonged SE-induced neuronal injury of pyramidal cells layers in CA1 and CA3, as well as in the granular cell layer of DG region, of the hippocampal formation. In addition, we observed a significant reduction of TUNEL positive cells with Parawixin2 treatment at 24 h after SE.

Another outcome analyzed in this work was reactive gliosis. The hallmark of reactive gliosis in neuronal injury is characterized by hypertrophy of cellular processes of astrocytes and upregulation of intermediate filament proteins, mainly GFAP [35]. These cells, when facing an insult present enlarged soma size, longer projections and increased GFAP expression [36], therefore, this feature indicates that GFAP can be used as a potential biomarker of neurotoxicity after brain injury [37,38,39]. Parawixin2 treatment also significantly decreased the reactive astrocytosis seen 24 h after SE. Higher levels of GFAP+ cells were observed in the hippocampal formation of rats that received vehicles after SE, however, animals treated with 0.86 μM Parawixin2 showed less GFAP+ labeling. All other treatments exhibited a moderate degree of GFAP+ cells.

Noteworthy, Parawixin2 is still neuroprotective in hippocampal cell layers 72 h after SE. We observed that animals treated with Parawixin2 showed higher cell density, and reduction of TUNEL and GFAP+ cells when compared to the vehicle group. According to Fujikawa (1996), both GFAP and TUNEL labeling presents a progressive rise in cells through time, even though more extensive death is observed at 72 h [32].

We attributed this neuroprotective effect of Parawixin2 to its inhibitory activity on GABA and glycine uptake, as previously reported by our group [24]. Enhancement of GABA transmission by inhibition of GABA uptake has gained much attention as a therapeutic strategy since it functionally increases the effect of GABA in a use-dependent manner, and GABA uptake inhibitors have proved to be effective as anticonvulsants in a variety of experimental models of epilepsy and in human epilepsy [40]. Considering that GABAergic neurotransmission plays an important role in the generation and maintenance of epileptogenesis and emerges as a potential target for therapeutic intervention, compounds that act on the inhibitory neurotransmission may be important in the study of epilepsy [41,42,43,44,45,46].

GABA clearance from the synaptic cleft is conducted by four types of GABA transporters: GAT-1–GAT-3 (high affinity) and BGT-1 (low affinity) [47]. Inhibitors of GAT-2, GAT-3, and BGT-1 are currently considered as potential drug targets for treatment of epilepsy [23] as they counterbalance the decreased calcium-independent GABA release, which is one of the main features in TLE hippocampus [48].

Comparing the effect of Parawixin2 with nipecotic acid, we observed that there was a higher degree of neuroprotection with Parawixin2. The highest dose of Parawixin2, which resulted in the most neuroprotection, is 100 times lower compared to the concentration of nipecotic acid used in this study (93 µM). Interestingly, co-administration of Parawixin2 at 0.43 µM with nipecotic acid resulted in a significantly higher protection, when compared to groups that received 0.43 µM Parawixin2 or nipecotic acid alone. Nipecotic acid is an inhibitor of GAT-1 and GAT-3 [49], which are localized in both glial and neurons [50]. On the other hand, Parawixin2 inhibits GAT-1 transport in transfected COS-7 cells, but it does not act on GAT-3 (unpublished data). Therefore, it is plausible to speculate that the co-treatment of these two drugs resulted in a synergistic effect, potentiating the GAT-1 inhibition.

The co-treatment of Parawixin2 with riluzole exerted a more potent neuroprotection effect in comparison to the co-treatment with nipecotic acid. This greater effect could be attributed to the complementary effect of the two compounds, as in addition to GABA transporters inhibition, riluzole blocks pre and post-synapse glutamatergic neurotransmission [51]. Therefore, we cannot rule out riluzole secondary effects in the observed neuroprotection. Complimentary to this idea, the inhibitory action of Parawixin2 on the glycine transporters may be partly responsible for the neuroprotective effects observed in this work. Glycine acts on ion channels activated by its binding to receptor sites of high-affinity ligand [52]. Additionally, the removal of glycine from synaptic terminals is accomplished by high-affinity transporters, which often recognize both GABA and glycine [16]. GABA and glycine transporters are targets for a wide range of therapeutic drugs used in the treatment of psychiatric diseases, including major depression, anxiety disorders, attention deficit hyperactivity disorder and epilepsy [53].

In the past, GABA uptake inhibitors development led to the production of classical drugs, such as nipecotic acid and guvacine, as central components of the derivatives containing lipophilic diaromatic side chains on the amino group of these cyclic amino acids [54,55,56]. However, among several compounds produced, few drugs acting on GAT (GABA transporters) subtypes are available for therapeutic use [40].

Currently, the most studied GABA transport inhibitors are nipecotic acid and Tiagabine [57]. However, nipecotic acid has a poor Blood-Brain Barrier penetration profile [58]. Tiagabine, marketed as Gabitril^®^ [59], is currently approved for treatment of partial seizures and is the only GAT inhibitor used as treatment for epilepsy [60]. However, Tiagabine is not prescribed as treatment, but it is usually an add-on in polytherapy, i.e., therapy with two or more drugs used at the same time [61]. Additionally, some studies indicate that tiagabine can produce some side effects, as shown by reports of high doses of Tiagabine leading to electroencephalographic paroxysms and non-convulsive SE both on patients [62,63,64] and rats in experimental models [65]. Additionally, other side effects associated with Tiagabine use include agitation, sedation and psychotic-like episodes in patients predisposed to psychiatric illness [40].

On the other hand, Parawixin2 seems to have a good therapeutic profile, as we have not observed cognitive or locomotor impairment after administration of Parawixin2 at a much higher dose than its ED_50_, therefore demonstrating a lack of toxicity in this pre-clinical model [26]. Moreover, Parawixin2 can cross the Blood-Brain Barrier [24], different from nipecotic acid, and exhibits an anxiolytic-like effect in anxiety models [27].

## 4. Conclusions

This study shows that Parawixin2 has neuroprotective potential against severe hippocampal damage triggered by long-term SE, up to 72 h later. Not only did Parawixin2 treatment preserve the neuronal population in all hippocampal layers, but also reduced the gliosis; both central features associated in TLE. Parawixin2 treatment resulted in a higher level of neuroprotection compared to riluzole or nipecotic acid treatment, even when administered in a lower concentration. Co-treatment with Parawixin2 and these two compounds indicate that potentiating GAT-1 inhibition may constitute a relevant approach for therapy. This work highlights a therapeutic potential of Parawixin2 as a GABA uptake inhibitor.

## 5. Materials and Methods

### 5.1. Parawixin2

Parawixin2 was obtained as described in detail by Beleboni and co-workers [24] and modified by Godoy et al. [20]. Briefly, *Parawixia bistriata* spider specimens were collected according to the Brazilian Chico Mendes Institute for the Biodiversity Conservation (ICMBio- SISBIO protocol No. 46797), in the region of Ribeirão Preto (São Paulo, Brazil) and frozen at 20 °C. Tweezers and ophthalmic scissors were used to extracted glands and venom reservoirs from approximately 3000 spiders, which were macerated, homogenized in deionized water/acetonitrile (ACN; 9:1, *v*/*v*) and centrifuged at 10,621× *g* for 3 min at 4 °C. The supernatant was ultra filtered using a 2000 Da Microcon^®^ filter (Millipore, Billerica, MA, USA). The extract containing compounds with molecular weight lower than 2000 Da was collected, lyophilized, weighed and solubilized in 20 mL of H_2_O/ACN 9:1 (*v*/*v*). This solution was filtered through a cellulose acetate membrane (0.45 µm, Millipore, USA) and 1 mL aliquots (12 mg/mL) were injected into a HPLC-DAD coupled to a Shimadzu C-18 reversed phase column (250 mm × 20 mm, 5 µm spherical silica, Shimadzu Scientific Instruments, Rydalmere, NSW, Australia) for preparative scale fractioning. A second stage of isolation used an elution program infusing 0.05% trifluoroacetic acid (TFA)/H_2_O at 1.0 mL/min flow for 10 min.

The material obtained after fractionation was injected into a high-resolution electrospray ionization quadrupole/time of flight (ESI-Q/TOF) using syringe pump (Harvard Apparatus, Holliston, Massachusetts, USA), at a flow rate of 10 L/min. Spectrum were acquired on an UltrOTOF apparatus (Bruker Daltonics, Billerica, Massachusetts, USA). Collision-induced dissociation was performed on the isolated protonated molecule using N2 as collision gas and 15 eV as collisional energy.

Parawixin2 identity was confirmed by ESI-HRMS and ESI-MS/MS spectra, which were compared to the compound previously isolated. ESI-HRMS afforded [M + H]^+^ at *m*/*z* 175.1196, giving us the molecular formula C_6_H_15_N_4_O_2_ with a 0.6 ppm error. ESI-MS/MS analysis showed a key fragment ion at *m*/*z* 116 (loss of loss of NHCONH2) in addition to the neutral losses of NH3 (17 m.u.) and CONH3 (45 m.u.) as previous reported by either Beleboni et al. (2006) and Godoy et al. (2017). All ions were formed by neutral eliminations by charge retention mechanism (29) confirming the presence of the functional groups.

Purity was estimated by LC-UV-ESI-MS, which resulted no other ions except those produced by Parawixin2 by in source dissociation, and by directly infusion in ESI-MS source showing a single majority peak related to the protonated molecule.

Parawixin2 modulation of GABA transport activity was confirmed in synaptosomes from rat cerebral cortex, as previously described [20,24].

### 5.2. Animals

Male Wistar rats (250–270 g) were obtained from the central vivarium at the University of São Paulo (Campus of Ribeirão Preto). Rats were kept in pairs in polypropylene cages and housed in a maintenance vivarium with ventilation and temperature controlled (25 ± 2 °C) with light/dark cycle of 12 h (lights on 07:00 a.m.). Access to water and chow was unrestricted.

This work was approved by the Ethics Committee for Experimental Animals at the University of São Paulo Campus of Ribeirão Preto (CEUA; protocol # 06.1.605.53.8), that follows the Guidelines of the Brazilian College of Animal Experimentation; Guiding Principles for Research Involving Animals and Human Beings; American Physiological Society and Ethical Guidelines for Investigations of Experimental Pain in Conscious Animals and the Brazilian Federal Law # 11794, which regulates the procedures for scientific use of animals.

### 5.3. Surgery

Animals were previously anesthetized with ketamine (60 mg/kg; Agener União, Embu-Guaçu, São Paulo, Brazil) and xylazine (8 mg/kg; Calier, Barcelona, Spain), both injected intraperitoneally (i.p), for implantation of a stainless-steel guide cannula (10 mm length), in the right lateral ventricle. We followed stereotaxic coordinates according to rat brain atlas of Paxinos and Watson (1998) [66] (A.P.—0.9 mm; ML—1.6 mm; DV—3.4 mm, from bregma). Briefly, a cannula was attached to the skull with acrylic resin and was temporarily sealed with a stainless-steel wire to protect it from obstruction. Rats were left to rest for at least 5 to 7 days for surgery recovery before experimental procedures.

### 5.4. Chemicals

Pilocarpine hydrochloride and drug treatments were administered through the guiding cannula. Pilocarpine, a muscarinic receptor agonist, was used to induce Status Epilepticus. The drugs riluzole (RIL) and nipecotic acid (NIP), which are neuroprotective GABA uptake inhibitors [31] were used as comparative-positive control. All drugs were purchased from Sigma (St. Louis, Missouri, USA), freshly prepared by dilution in phosphate buffered saline (PBS, 0.05 M; pH = 7.4) and injected via intracerebroventricularly (i.c.v.) in a volume of 1 μL. Microinjection procedures were carried out with a 10 µL syringe (Hamilton, Reno, Nevada, USA) connected via a silicon tube to a 30 G stainless-steel needle, with 10.1 mm of length. A microinjection pump (Insight, Ribeirão Preto, São Paulo, Brazil) was used to drive the speed of injections (0.5 μL/min).

### 5.5. Pilocarpine Administration, Behavior Analysis and Drug Treatment

After surgery recovery, rats received a pilocarpine microinjection via i.c.v. (dose of 2.4 mg/µL, in a volume of 1 µL) for induction of SE. The SE was determined when animals had shown continuous tonic-clonic generalized seizures lasting for at least 30 min, without spontaneous recovery [32]. Behavioral seizures were scored according to Racine scale for limbic seizures [33].

After 180 min from the onset of SE, animals received one dose of thiopental sodium (30 mg/kg; i.p) to attenuate SE. Animals that ceased spontaneously SE before 3 h were discarded from the study (*n* = 12). The neuroprotective effects of Parawixin2 in hippocampal regions were analyzed at either 24 h or 72 h after SE.

Rats were randomly assigned to treatments 60 min after SE termination. Animals were treated by i.c.v. route either with vehicle (PBS) (SE + VEH *n* = 13), riluzole (SE + RIL *n* = 9) at 10 µM; nipecotic acid (SE + NIP *n* = 7) at 93 μM or different doses of Parawixin2, as follows: 0.21 μM (SE + Pwx2 0.21 μM; *n* = 5), 0.43 μM (SE + Pwx2 0.43 μM; *n* = 7) or 0.86 μM (SE + Pwx2 0.86 μM; *n* = 9). Additionally, animals received co-treatments with compounds, as follows: SE + RIL + Pwx2 0.43 μM (*n* = 5), and SE + NIP + Pwx2 0.43 μM (*n* = 5). The intermediate concentration of Parawixin2 (0.43 μM) was chosen to evaluate the potential synergistic effect of the co-treatment with two compounds. All treatments were injected in the final volume of 1 μL. Therefore, the molar concentration represents the number of molecules, which enables comparison between drugs regardless the molecular mass. All these animals were euthanized 24 h later (24-h groups). The remaining animals received vehicle (SE + VEH) or Parawixin2 0.86 μM (SE + Pwx2 0.86 μM) microinjections and were euthanized 72 h after the interruption of SE (72-h groups).

### 5.6. Tissue Preparation

Rats were euthanized with an overdose of sodium thiopental (120 mg/kg; i.p) either at 24 h or 72 h after SE induction. Anesthesia was followed by a transcardiac perfusion through the left ventricle with phosphate buffer (PBS 0.1 M, diluted in water; pH 7.4) followed by fixative solution of 4% paraformaldehyde (PFA, diluted in PBS 0.1 M, pH 7.4; Synth, São Paulo, São Paulo Brazil) for 15 min (flow of 20 mL/min). The control group underwent the same procedure, the perfusion period was counted from the injection of vehicle. Brains were post-fixed in 4% PFA (diluted in PBS 0.1 M) for a period of 12 h at 4 °C and cryoprotected with serial sucrose solutions (10%, 20% and 30% diluted in PBS 0.1 M) for a period of 24 h (4 °C).

### 5.7. Section of Brain Tissue

After cryoprotection, brains were covered by OCT (Tissue-Tek; Jung, Frankfurt, Germany), frozen in isopentane (Merck, Kenilworth, New Jersey, USA) previously cooled with dry ice, and kept in a freezer at −80 °C. The hippocampal formation (between −3.14 mm and −4.3 mm from bregma) [30] was sectioned in coronal 40 µm serial slices using a cryostat (Leica Microsystems, Heidelberg, Germany). The hippocampal slices (15 sections per animal) were stretched on glass slides previously gelatinized and afterward histological techniques were performed as described below.

### 5.8. Nissl Staining with Cresyl Violet

Nissl staining was used as an indicator of neuronal viability, as upon the occurrence of neuronal injury rough endoplasmic reticulum may disappear (a phenomenon known as chromatolysis) [34]. This histological technique was used to enable viewing of the pyramidal cell layers of CA1 and CA3, and granule cell layer of the dentate gyrus (DG) of the hippocampus, allowing the subsequent counting of viable neurons to estimate neuronal densities. Briefly, sections were dehydrated and slides were consecutively rinsed in 95%, 80% and 70% ethanol solutions (*v*/*v*, in distilled water). Then slides were then rinsed in water and in cresyl violet solution (0.5%) for 30 min and then washed with distilled water. Subsequently, sections were dehydrated by consecutive washes of increased concentrations of ethanol solution and xylene. Slides were covered with glass coverslips and Permount (Fisher Scientific, Hampton, New Hampshire, USA) as a mounting medium.

### 5.9. Glial Fibrillary Acid Protein (GFAP) Immunostaining

The protocol used to GFAP immunolabelling was performed as described by Cunha and co-workers [35]. Briefly, slides were washed in PBS, immersed in glycine (0.1 M, diluted in 0.05 M PBS; Sigma, St. Louis, Missouri, USA) for 5 min and right after were covered with bovine serum albumin (1% BSA diluted in 0.05 M PBS; Sigma, Missouri, USA) for 30 min at 37 °C. Then, the tissue was incubated with primary antibody against GFAP (mouse monoclonal antibody, dilution 6:1000 in 0.05 M PBS; Dako, Glostrup, Denmark) for 120 min at 37 °C. Afterward, slides were washed in PBS and incubated with secondary antibody fluorescein isothiocyanate (FITC—anti-mouse IgG; dilution 20:1000, *v*/*v* in 0.05 M PBS; Dako, Glostrup, Denmark) for 30 min at 37 °C in a dark room. All antibodies were diluted 30 min before the experiment and previously centrifuged at 10,000× *g* at 4 °C for 10 min. The slides were then washed in PBS, covered by a glass coverslip using glycerol (diluted in PBS 9:1, *v*/*v*) as the mounting medium and immediately examined under a fluorescent microscope (DM 5000B Leica Microsystems, Heidelberg, Germany).

### 5.10. Terminal Deoxynucleotidyl Transferase Immunostaining for Biotin-dUTP-Nick-End-Labeling (TUNEL)

For immunohistochemical assays, the sections were incubated in a solution of 4% PFA (diluted in PBS 0.05 M) for 15 min, then placed in PBS (0.05 M) for 15 min, always at room temperature. Subsequently, sections were covered with proteinase K (2 mg/mL, Upstate, Lake Placid, New York, USA) diluted in Tris (10 mM, pH 8) at room temperature for 10 min. After that, slides were washed with PBS and TdT-buffer (125 μM Tris-HCl, 1 M sodium cacodylate, 1.25 mg BSA/mL, 1 mL of deoxynucleotide-terminal-transferase-TdT and 0.5 mL of fluorescein-12-dUTP, pH 6.6; Upstate, USA) for 15 min at room temperature. Posteriorly, slides were incubated with TdT-end-labeling (TdT-buffer, biotin-dUTP and TdT enzyme in a ratio of 90:5:5, *v*/*v*) in a moist chamber at 37 °C for 1.5 h. After this period, the sections were washed in PBS and blocked for 20 min at room temperature and the slides were incubated in Avidin-FITC diluted in blocking solution (ratio 1:9 *v*/*v*; Upstate, USA) at 37 °C for 30 min in the absence of light. Finally, slides were washed in PBS and covered with a glass coverslip using glycerol (diluted in PBS 9:1, *v*/*v*) as the mounting medium.

### 5.11. Analysis of Neuronal Damage and Immunofluorescence

Images of the entire length of the pyramidal cell layer of CA1, CA3, and the granule cell layer of the dentate gyrus (DG) with a digital color camera (DFC 300 FX, Leica Microsystems, Heidelberg, Germany) connected to a microscope (DM 5000 B, Leica Microsystems, Heidelberg, Germany) and a computer, using bright field for Nissl staining and FITC filter for GFAP and TUNEL labeling. The images for cell quantification and area measurement were captured using a 40× objective, while the images obtained with a lens of 100× magnification was used to measure the diameter of the neuronal nuclei. The viability of neurons and measurement of hippocampal areas were blindly performed by a well-trained investigator, using the software Q-Win (Leica Microsystems, Heidelberg, Germany). The number of viable cells was determined by averaging the mean values obtained through the analysis of three different sections of the hippocampus. The actual number of cells was calculated using the Abercrombie correction method [36]. Values were expressed as the mean densities (± SEM).

For the quantification of GFAP reactive cells (GAFP+) a semi-quantitative method was used, according to Drage and co-workers [67]. Immunoreactive astrocytes were considered only those with more than three extensions radiating from a single nucleus. The amount of (GFAP+) cells was measured using a scale of values mean on the activity of astrocytes in the pyramidal cell layers of CA1 and CA3, as well as in the granule cell layer of the DG of the rats. The values of this scale were characterized as grades 1, 2 and 3 referring to “weak,” “moderate” or “intense,” respectively. This classification considered the average amount of reactive cells observed in three separate sections of each region for individual analysis.

To quantification of TUNEL positive (TUNEL+) cells was performed using a range of semi-quantitative measurement, according to Martinez and colleagues [68], in the same hippocampal areas assessed for GFAP. This scale used the higher quantity of TUNEL labeled cells observed in this study as a guideline, depicted as 0 (no reaction), 1 (low reactivity), 2 (moderate reactivity) and 3 (intense reactivity). All immunoreactive cells were quantified regardless of the intensity of fluorescence.

### 5.12. Statistical Analysis

Histological quantification was analyzed using one-way ANOVA followed by post hoc Student–Newman–Keuls test. A two-way ANOVA followed by the post hoc Sidak test was used to analyze the effects of time and treatment with Parawixin2. Results are shown as means ± standard error of the mean (SEM). All data analyses were performed using GraphPad Prism 6 Software (GraphPad Software Inc., San Diego, CA, USA).

## Figures and Tables

**Table 1:** Grade of TUNEL immunoreactive cells in the hippocampal regions CA1, CA3, and DG 24 h and 72 h after long-lasting pilocarpine-induced SE.

Experimental Groups (*n* = 5–9)	TUNEL+ Cells		
	CA1	CA3	DG
VE + VEH	0.11 ± 0.11 ***	0.0 ± 0.0 **	0.0 ± 0.0
SE + VEH	2.0 ± 0.26	2.0 ± 0.22	1.2 ± 0.37
SE + RIL	1.3 ± 0.21	1.3 ± 0.42	0.4 ± 0.25
SE + NIP	1.8 ± 0.37	1.4 ± 0.51	0.6 ± 0.25
SE + Pwx 0.21 μM	1.5 ± 0.34	1.3 ± 0.33	1.0 ± 0.26
SE + Pwx 0.43 μM	1.0 ± 0.37	1.3 ± 0.25	0.3 ± 0.21
SE + Pwx 0.86 μM	0.8 ± 0.37 *	0.3 ± 0.25 *	0.2 ± 0.17
SE + RIL + Pwx 0.43 μM	1.7 ± 0.21	0.3 ± 0.33 *	0.3 ± 0.22
SE + NIP + Pwx 0.43 μM	1.8 ± 0.37	1.7 ± 0.33	0.7 ± 0.21

The data represent the values mean (± SEM) of a semi-quantitative scale. Quantification of TUNEL reactive cells using a semi-quantitative scale (0 = no reaction, 1 = low reactivity, 2 = moderate reactivity and 3 = intense reactivity). * *p* < 0.05, ** *p* < 0.01 and *** *p* < 0.001, compared to group SE + VEH (one-way ANOVA followed by Student–Newman–Keuls post-test).
